# Additional Marketing Responses to a Tax on Sugar-Sweetened Beverages: Comment on "Understanding Marketing Responses to a Tax on Sugary Drinks: A Qualitative Interview Study in the United Kingdom, 2019"

**DOI:** 10.34172/ijhpm.2022.7638

**Published:** 2022-11-14

**Authors:** Jasmina Ilicic, Stacey Brennan, Katherine Cullerton

**Affiliations:** ^1^Monash Business School, Monash University, Caulfield East, VIC, Australia.; ^2^The University of Sydney Business School, The University of Sydney, Sydney, NSW, Australia.; ^3^School of Public Health, University of Queensland, Herston, QLD, Australia.

**Keywords:** Soft Drink Levy, Tax, Marketing, Health Policy, Soda

## Abstract

Marketing responses to sugar-sweetened beverage (SSB) taxes are understudied in the literature. Previous research has been limited to examining price and advertising, in particular promotions responses. Forde et al advocate for a focus on exploring a range of marketing responses to a SSB tax, with an emphasis on the marketing mix (price, promotion, product, and place). Their qualitative findings from the United Kingdom focus mostly on possible product and price decisions, with limited discussion of place and promotions decisions. We argue that the proposed marketing mix decisions may be used to avoid or side-step a SSB tax and that their likelihood of adoption may be dependent upon additional factors besides brand strength, reputation, and portfolio size highlighted by Forde and colleagues, such as organizational capabilities, industry competition, and brand positioning. We recommend future research examine the importance of consumer behaviour in developing marketing programs and in response to the marketing mix levers pulled by industry.

## Introduction

 Recently, Forde and colleagues conducted a qualitative study investigating the potential marketing responses to the 2019 soft drinks industry levy (SDIL) on introduced in the United Kingdom.^1^ Through a series of interviews with academic, industry, and civil stakeholders, the authors reported on a number of approaches that spanned the marketing mix, that is; product, price and to a lesser extent place and promotion decisions. Forde and colleagues’ work identifies a number of ‘levers’ marketers can use to either embrace (eg, price increase), avoid (eg, product reformulation), or side-step (eg, decrease product size, change message tactics) the new tax. Forde’s research is important as it contributes to our understanding of potential organizational responses. We suggest that consideration should also be made on the likelihood that the strategies will be adopted, along with the downside of the strategies for both consumer behavior and the company’s bottom line.

 To understand the likelihood of strategy implementation, it is important to understand the context of the sugar-sweetened beverages (SSBs) industry. While global SSB consumption is declining, in the United Kingdom the soft drink industry is in a stage of growth with forecast sales of 10 026 million litres in 2021 to 10 298 million litres in 2026.^2,3^ The soft drink industry in the United Kingdom is dominated by four key players. *Coca-Cola* is the leading company by market share, followed by *Pepsi-Cola*, *Britvic*, and *Suntory Holdings*, respectively. *Coca-Cola* and *Pepsi Co Inc* (Britvic Soft Drinks) alone account for almost two-fifths of the United Kingdom’s soft drink market.^3^ These companies are well established and maintain an extensive product portfolio that contain both high and low sugar alternatives.

## Is Product Reformulation a Likely Response?

 Interestingly, an intention to avoid the levy through product reformulation was the most cited response within Forde and colleagues’ research. It is suggested that marketers may seek to change their product offering by reducing the sugar content of SSBs, enabling their offering to be tax exempt. However, they do highlight that brands with larger portfolios are able to spread the risk and have less incentive to reformulate, especially is if they already offer low sugar alternative products. We suggest that changes to product offerings can be fraught with danger. We need only to look at *Coca-Cola*’s decision to alter its product formula in the 1980s to create ‘*New Coke*.’^4^ This product reformulation resulted in significant consumer backlash, including customer complaints and product boycotts. Although this reformulation occurred in response to an external competitive force, rather than a government force (eg, SDIL), it is important to recognize that implementing a product reformulation strategy relies on considerable research and development, from the perspective of both production and consumer effects. It is evident that “*… companies rarely reformulate existing products, but instead develop new, reformulated alternatives*.”^5^ When looking at the UK soft drink market it is apparent that the four key players in the market had adopted this strategy prior to the 2019 tax, maintaining a diverse range of products, creating a house of brands (numerous brands under a corporate brand portfolio that are independent of one another, and each with its own target market and marketing mix; eg, *Suntory Holdings*: *V* energy drink, *Lucozade, Suntory Tennensui* mineral water; *Coca-Cola*: *Coca-Cola*, *Powerade ION4*, *Pump*). The industry response appears to be to side-step the tax by introducing new products into the market. This offers consumers more choice and possible substitution brands within the same corporate brand portfolio, resulting in retention of customers and revenue.

## Is Portion Size Reduction a Likely Response?

 Forde and colleagues’ findings suggest that reducing portion size is a possible ‘product’ marketing response to the SDIL. They suggest that this decision may encounter less consumer resistance than reformulation since it does not pass the psychological threshold or just noticeable difference for consumers. However, recent data from the United Kingdom illustrates that in response to coronavirus disease 2019 (COVID-19) and consumers spending more time at home in remote work/study, companies are investing heavily in increasing portion sizes through the introduction of larger package sizes and multipacks to cater to the take-home market.^6^ For example, in 2021 energy drink brand *Lucozade* launched a 1450 mL pack sized bottle and a multipack of 12 × 330 mL cans of *Lucozade Energy*. Furthermore, following the introduction of a tax on SSBs in South Africa in 2019 companies implemented the largest increases in price for the smallest container carbonates (100% pass-through rate to consumers, that is, changes in the price after imposition of the tax), while price increases for larger containers were significantly smaller (50% pass-through rate to consumers).^7^ As such, companies can side-step the financial implications of the tax by introducing larger package sizes with smaller price increases, combining both product and price strategic responses to the tax. While it may appear in the short-term that companies will lose profits by offering these products with smaller pass-through tax rates, they are more likely to retain loyal customers who ultimately offer customer lifetime value (that is, repeat purchases across the lifetime of the customer) and, as such, profits in the long term. This product strategy has important implications for consumers who may switch to purchasing SSBs in larger package sizes or in multipacks, which represent better value for money, resulting in increasing their consumption volume of SSBs, and ultimately reducing the effectiveness of the SDIL on consumption behaviour.

## Is Changing to a Health or Heritage Messaging a Likely Response?

 Forde et al note that companies can alter their communications to focus on health or heritage following the introduction of the SDIL. Their discussion emphasizes changing the overall essence of the brand’s communications to consumers and policy-makers, rather than just changing communications through advertising campaigns. We suggest that this change in messaging is arguably focused on communicating a brand’s positioning. Brand positioning “*is the act of designing the company’s offering and image to occupy a distinctive place in the mind of the target market*.”^8^ The key to brand positioning is differentiating the brand so that it is perceived to offer better value than competing brands, resulting in a unique selling proposition.

 While Forde and colleagues’ findings suggest that companies could respond to the SDIL by focusing on a health-based positioning, this strategy is limited as it is easily copied, reducing competitive points of difference. Health is a form of direct benefit positioning, meaning that attributes (eg, low sugar) of the product that form the basis of the positioning strategy offer a direct health advantage.^9^ SSB brands, however, are more likely to differentiate themselves from their competitors in terms of indirect benefit positioning, based on the brand satisfying experiential or symbolic needs of consumers.^10^ For example, *Coca-Cola* had previously positioned itself on the experiential need of joy or happiness (“Open Happiness”). In 2021 the brand shifted its global brand positioning to emphasize symbolic needs, focusing on social connections while altering the brand logo to represent a hug (“Real Magic”). *Pepsi*, on the other hand, has long positioned itself on symbolic needs of youthful individuality and self-actualization, lending associations tied to celebrities such as Cardi B, Michael Jackson, and Beyonce, and partnerships with sport and sport stars such as UEFA Champions League’s Lionel Messi and Ronaldinho in 2022 (“Play to Inspire”). As such, we suggest that brands are unlikely to focus on positioning their brand based on the attribute of health, which does not offer a competitive advantage. We argue that it is more likely that brands would aim to differentiate themselves from competitors based on experiential or symbolic benefits the brand provides to consumers.

 Furthermore, Forde and colleagues suggest that well established and strong brands in the market could focus on their brand authenticity, in terms of their heritage or continuity. However, we suggest that there is high similarity in heritage of the key players in the market (eg, *Coca-Cola*, *Pepsi*). As such, focusing on brand authenticity is an unlikely response to the SDIL as it focuses on points of parity not on points of difference between brands, which would not offer a competitive advantage to these brands.

## But What About Consumer Behavior?

 The theoretical framework developed by Forde and colleagues captures the strategic marketing process, which is based on the model of value creation.^11^ Based on this five-stage model, marketers need to:

- understand the market in terms of the internal and external marketing environment and consumer behaviour;
- design a customer-driven marketing strategy through segmentation (dividing the market into smaller markets), targeting (selecting the segment/s to enter), and positioning;
- develop an integrated marketing program that delivers superior value (marketing mix);
- build profitable relationships (retaining current customers and building lasting customer relationships); and
- capture value from customers to create profits and customer equity.

An important step in this process not captured by Forde and colleagues’ model is target marketing, which is based on developing product, pricing, promotions, and place decisions tailored to each targeted segment (stage *ii* and *iii*).

 Previous research has shown that certain segments of the market, that is, sociodemographic (age/socioeconomic status) and behavioral (usage rate) will respond differently to a SSB tax. For example, SSB taxes are more effective at reducing consumption intentions in adults than in children.^12^ These findings suggest that marketing responses to SSB taxes are likely to be targeted to adults only, which may continue the detrimental effects that excessive soft drink has on childhood health, for example, increasing dental caries.^13^ Furthermore, research has found that individuals respond differently to SSB taxes based on their behavior in terms of their current usage rate. High-consuming individuals are reported to be less responsive to price changes following a SSB tax.^14^ Moreover, it appears that a SSB tax is likely to have the greatest health gain impact for low socioeconomic groups, who subsequently also experience the greatest cost.^14^ Future research is needed to examine how marketers’ knowledge of segment differences (demographic, behavioral, and psychographic factors) influence their target marketing and the development of their marketing program in response to SSB taxation.

 While Forde and colleagues’ theoretical model incorporates “purchasing of soft drinks” as its dependent variable, there is little discussion of the influence of each of the marketing mix factors on consumer behavior. In other words, little is known regarding the effect of the marketing program on consumers (stage *iii*, *iv*, and *v* of the model of value creation). Previous research suggests that anti-SSB advertising messages can lead to psychological reactance, resulting in a negative attitude towards reducing SSB consumption and to any policies limiting an individual’s ability to consume SSBs.^15^ This relationship was amplified for high SSB consumption individuals and those with a conservative political orientation (another possible segmentation base that marketers need to consider in the development of their marketing mix). This study, however, was limited to public service announcement advertising only and intentions to consume SSBs, not on actual consumption behaviors. Future research is needed to examine the effects of the marketing mix factors independently (and in combination) on consumer behavior in terms of brand loyalty and actual purchase of soft drinks.

## Conclusion

 Forde and colleagues’ research contributes to our understanding of marketing responses to the introduction of levies on SSBs. This is particularly relevant given that research into marketing responses has received limited attention from both academics and policy makers alike. As suggested by Forde and colleagues, marketers have a number of levers that can be used when faced with a change in market conditions, such as the introduction of a SSB tax. These levers can include changes their product, price, placement, and/or promotion strategies. We suggest additional levers can be used such as altering their approach to segmentation, targeting, and positioning. While Forde et al present potential strategies, limited consideration was given to the feasibility of the approaches highlighted and that companies do not consider any one response in isolation. We argue that while the proposed strategies could be used to avoid or side-step changes in the external marketing environment (eg, SDIL), their likelihood of adoption may be dependent upon additional factors beyond brand strength, reputation, and portfolio size such as organizational capabilities, industry competition, and brand positioning. We propose future research examine the full strategic marketing process, or model of value creation, specifically examining the importance of consumer behavior in developing marketing programs and in response to the marketing mix levers pulled by industry.

## Ethical issues

 Not applicable.

## Competing interests

 Authors declare that they have no competing interests.

## Authors’ contributions

 Conception and design: JI, SB, and KC; Analysis and interpretation of data: JI and SB; Drafting of the manuscript: JI, SB, and KC; Critical revision of manuscript: JI, SB, and KC.
